# Inhibitory Immune Checkpoint Receptors and Ligands as Prognostic Biomarkers in COVID-19 Patients

**DOI:** 10.3389/fimmu.2022.870283

**Published:** 2022-03-31

**Authors:** Mohammad A. Al-Mterin, Alhasan Alsalman, Eyad Elkord

**Affiliations:** ^1^ Natural and Medical Sciences Research Center, University of Nizwa, Nizwa, Oman; ^2^ Biomedical Research Center, School of Science, Engineering and Environment, University of Salford, Manchester, United Kingdom

**Keywords:** SARS-CoV-2, COVID-19, inhibitory immune checkpoints, ligands, prognostic biomarker

## Abstract

Coronavirus disease 2019 (COVID-19) is caused by SARS-CoV-2. During T-cell activation, the immune system uses different checkpoint pathways to maintain co-inhibitory and co-stimulatory signals. In COVID-19, expression of immune checkpoints (ICs) is one of the most important manifestations, in addition to lymphopenia and inflammatory cytokines, contributing to worse clinical outcomes. There is a controversy whether upregulation of ICs in COVID-19 patients might lead to T-cell exhaustion or activation. This review summarizes the available studies that investigated IC receptors and ligands in COVID-19 patients, as well as their effect on T-cell function. Several IC receptors and ligands, including CTLA-4, BTLA, TIM-3, VISTA, LAG-3, TIGIT, PD-1, CD160, 2B4, NKG2A, Galectin-9, Galectin-3, PD-L1, PD-L2, LSECtin, and CD112, were upregulated in COVID-19 patients. Based on the available studies, there is a possible relationship between disease severity and increased expression of IC receptors and ligands. Overall, the upregulation of some ICs could be used as a prognostic biomarker for disease severity.

## Introduction

Coronavirus disease 2019 (COVID-19) is a pandemic disease from December 2019 (1). Since the initial wave of cases appeared in Wuhan, China, over 260 million individuals worldwide have been infected with COVID-19, resulting in about six million deaths until now. Most infected patients are without any symptoms or have mild symptoms, but some patients become severely ill and need to be admitted to the hospitals (1, 2). This unexpected outbreak has highlighted the necessity to develop new vaccinations and different therapies to combat COVID-19 (3). Importantly, there are new approved direct antiviral medications for COVID-19 patients (4). For example, remdesivir, a nucleoside analog, is incorporated into the SARS-CoV-2 viral RNA-dependent RNA polymerase (RdRp) complex and prevents its translocation (5). The US Food and Drug Administration (FDA) has authorized it for the treatment of hospitalized COVID-19 patients (5, 6). Furthermore, molnupiravir, a nucleoside analogue, is the first orally taken direct-acting antiviral drug that has been shown to be effective in the eradication of viral RNA, while maintaining high safety and tolerability profiles (4, 7).

COVID-19 could be an immune-related disorder, characterized by lymphopenia, increased proinflammatory cytokines, and abnormal T-cell responses (3, 8, 9). It can stimulate both innate and adaptive immune responses. Later, this causes severe inflammatory reactions leading to systemic cellular damaging (10). However, the transition from innate to adaptive immune responses is crucial in defining the clinical implications of COVID-19 infections. First responses are often protective, whereas later leads to a reduction in viral clearance and a low survival rate (8, 11). Tissue injury observed in acute COVID-19 infections is mediated primarily by the hyperreactivity of lymphocyte responses (8).

## T Cells in COVID-19 Patients

Lymphopenia is a general characteristic of many respiratory viral diseases such as human rhinovirus and influenza (12). COVID-19-associated lymphopenia could be more severe and persistent, compared with other respiratory infections (12, 13). Although lymphopenia is not fully understood in COVID-19, the decline in T-cell numbers is a common symptom among patients with severe diseases (14). Recent studies showed a decline in the total number of T cells, as well as a negative relationship between T-cell depletion and prognosis, particularly in COVID-19 patients who require admission to the ICU (15, 16). Moreover, COVID-19 can be more severe in patients who arrive at the hospital with low CD4+ and CD8+ T cell numbers, which can lead to worse clinical outcomes (17). Clearly, these patients should be monitored for any changes in levels of T cells (18). In severe cases of COVID-19, it has been shown that CD8+ T cells and natural killer (NK) cells were reduced in numbers, but they were hyperactive (19). The number and immunological status of GrA+CD8+ T cells and NK cells were recovered after the patients’ condition improved (19). According to this study, perforin+ NK cells and GrA+CD8+ T cells could be useful for the diagnosis of COVID-19 patients. Memory T cells are essentially important to fight against SARS-CoV-2 reinfection and to determine the duration of vaccine protection (20). A study demonstrated that virus-specific T cells induced by betacoronaviruses are long-lasting, suggesting that COVID-19 patients will develop a long-term T-cell immunity, which may be able to protect against SARS-CoV-2 (21). In addition, Odak et al. found that hospitalized COVID-19 patients showed altered effector/effector memory and naïve T-cell frequencies, compared with healthy controls (22). Also, they found that T regulatory cells were significantly lower in both severe and mild COVID-19 patients, compared with healthy controls (22). Moreover, they observed increased levels of effector and memory T-cell populations in mild disease but not in severe disease (22).

## Inhibitory Immune Checkpoints in COVID-19

During T-cell activation, the immune system uses checkpoint pathways to maintain co-stimulatory and co-inhibitory signals. As a result, a disturbance in the function of ICs may lead to autoimmune diseases. Some cytokines regulate the expression of immune checkpoint proteins. As an example, transforming growth factor-β1 (TGF-β1) increases the expression of the programmed cell death-1 (PD-1) receptor by enhancing antigen-driven PD-1 gene transcription through Smad3 transcriptional activation in T cells *in vitro* and in tumor-infiltrating lymphocytes *in vivo* (23). Moreover, Schlichtner et al. found that VISTA upregulation is regulated by the TGF-β1-Smad3 signaling pathway (24). However, in T cells, TGF-β regulate the expression of VISTA only on T cells lacking granzyme B expression (24). Indeed, they also reported that TGF-β may regulate galectin-9 (Gal-9) expression by the Smad3 pathway in tumor cells (24).

Many pathogens are able to induce overexpression of these checkpoint molecules in different immune cells, leading to increases in IC inhibitory signals and immune evasion (25, 26). As a consequence of IC expression, T cells are exhausted, leading to viral escape from immune monitoring (26, 27). **Table 1** summarizes IC receptors and ligands covered in this review.

**Table 1:** Summary of immune checkpoint receptors (A) and ligands (B) covered in this review.

A		B	
IC receptors	Cellular expression pattern	Ligand	Cellular expression pattern
PD-1	T cells, NKT, NK, B cells, activated monocytes, DCs.	PD-L1	Hematopoietic and non-hematopoietic cells.
CTLA-4	T cells	PD-L2	DCs, macrophages, peritoneal B1 cells, helper T cells, bone marrow-derived mast cells.
LAG-3	T cells, NK, B cells.	Galectin-9	T cells, B cells, macrophages, mast cells.
TIM-3	T cells, DCs, macrophages, monocytes.	CD112	DCs, monocytes.
TIGIT	T cells, NK, Tregs.	CD155	DCs, monocytes.
BTLA	T cells, B cells, DCs, monocytes.	B7-H3	DCs, monocytes, T cells, B cells, NK.
VISTA	T cells, myeloid cells.	LSECtin	Myeloid cells.
2B4	T cells, NK, DCs, monocytes.	Galectin-3	Macrophages, monocytes, DCs, eosinophils, mast cells, NK, activated T and B cells.
NKG2A	T cells, NK.		
CD160	T cells, NK, NKT.		

### Programmed Cell Death-1

PD-1 works by inhibiting innate and adaptive immune responses (28, 29). It is expressed on B cells, T cells, activated monocytes, natural killer T (NKT) cells, natural killer cells (NK), and dendritic cells (DCs) (28, 30–33). PD-1 modulates T-cell function and tolerance, as well as immune-mediated tissue injury (34, 35). There are two known ligands for the PD-1 receptor: PD-L1 and PD-L2. In normal circumstances, the PD-1/PD-L1 pathway plays a crucial role in the modulation of immune function and preventing autoimmunity by inhibiting T-cell activation (34, 36, 37). PD-1 is elevated during acute and chronic viral diseases, such as HCV, HBV, or HIV (31, 38). T-cell depletion and disease progression are linked to PD-1 expression in HIV-specific CD4+ and CD8+ T cells (31, 32, 38, 39).

**Figure 1:**
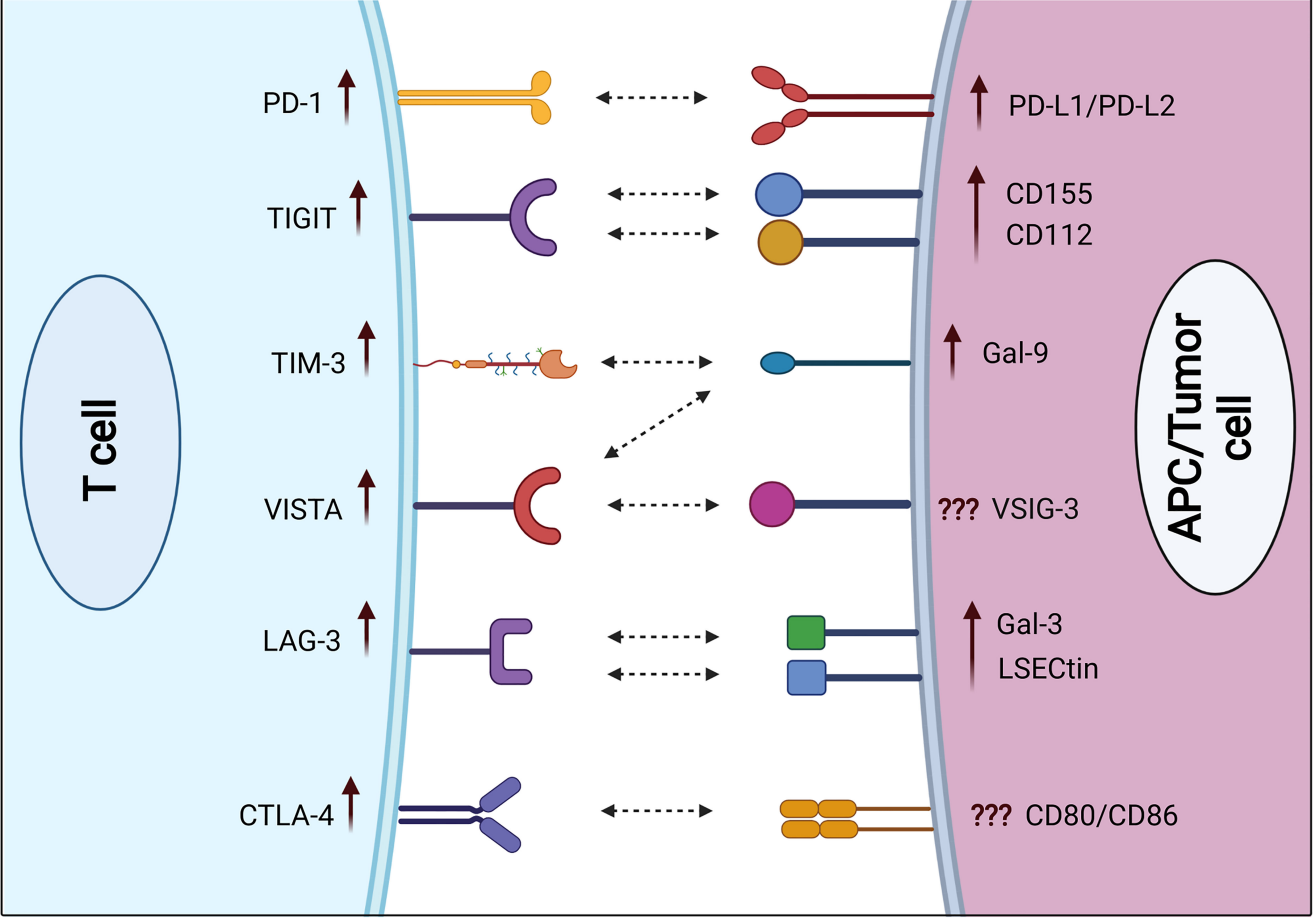
Expression of immune checkpoint receptors on T cells and their respective ligands on APCs and/or cancer cells in severe COVID-19 patients. Some ICs including PD-1, TIGIT, TIM-3, VISTA, LAG-3, and CTLA-4 are upregulated on both CD4+ and CD8+ T cells in severe COVID-19 patients, compared with mild/moderate patients. Various IC ligands including PD-L1, PD-L2, CD155, CD112, Gal-9, Gal-3, and LSECtin are upregulated on APCs in severe COVID-19 patients.

In COVID patients, PD-1 was shown to be overexpressed on both peripheral blood CD4+ and CD8+ T cells, compared with healthy controls (40). Some studies indicate that PD-1 is thought to have a role in T-cell exhaustion and disease progression (31, 39, 41, 42). The observed PD-1 expression was higher in peripheral blood CD4+ and CD8+ T lymphocytes in COVID-19 patients of all ages, compared with healthy controls (26, 39, 43). PD-1 was found to be upregulated on both peripheral blood CD8+ and CD4+ T cells in severe compared with mild and moderate diseases (**Figure 1**) (40, 43). Moreover, Kong et al. observed a significant increase in serum levels of soluble PD-1 (sPD-1) in severe COVID-19 patients, compared with mild disease (44). Jeannet et al. reported that the expression of PD-1 was increased exponentially with the period of illness in COVID-19 patients in the ICU, thus reducing the effectiveness of immune responses to viral infections (45). However, according to Rha et al., peripheral blood CD8+ T cells expressing PD-1 during COVID-19 infection are not exhausted but rather functional (46). In line with these findings, Shahbaz et al. found that the overexpression of PD-1 in peripheral blood was not associated with exhaustion and impairment of T-cell function (40).

### Cytotoxic T Lymphocyte-Associated Antigen-4

Cytotoxic T lymphocyte-associated antigen (CTLA-4) works by suppression of T-cell stimulatory signals (47–49). It binds both B7 family members (B7-1 and B7-2) with considerably higher affinity than CD28 (50). As a result, the CD28 receptor is excluded from the immunological synapse (50). This receptor also sends inhibitory signals to T cells, limiting their activation and finally leading to the depletion of its ligands *via* endocytosis on antigen-presenting cells (51, 52). CTLA-4 is hypothesized to control T-cell proliferation early in the immune responses, mainly in lymph nodes, while PD-1 inhibits T cells later, mainly in the peripheral tissues (53, 54).

Zheng et al. showed that the increased expression of CTLA-4 in severe symptomatic COVID-19 patients leads to CD8+ T-cell exhaustion in peripheral blood and impairs their specific immune activity (43). Moreover, Kong et al. found a significant increase in serum levels of soluble CTLA-4 (sCTLA-4) in severe COVID-19 patients compared with mild disease (44). Another study found that the upregulation of CTLA-4 in blood and bronchoalveolar lavage fluid (BALF) CD8+ and CD4+ T cells is due to viral invasion and excessive immune responses (55). Some recent studies reported that CTLA-4 is upregulated in peripheral blood CD8+ T cells in severe disease, compared with mild and moderate diseases (**Figure 1**) (40). Moreover, a specific upregulation of CTLA-4 was seen in BALF CD8+ T cells isolated from severe COVID-19 patients (56). Hou et al. showed that the expression of CTLA-4 on CD4+ T cells was dramatically elevated in patients with COVID-19 after 1 year of recovery (57). A study indicated that the presence of CTLA-4 and PD-1 on T cells was not associated with a T-cell inhibition, but rather with a strong activation (40). Other studies demonstrated that the presence of CTLA-4 and PD-1 on T cells may modulate the immune response and protect the vital organ from an excessive inflammatory environment in severe COVID-19 patients (51, 58).

### T-Cell Immunoglobulin and Mucin Domain-Containing Protein 3 and Lymphocyte-Activation Gene 3

T-cell immunoglobulin and mucin domain-containing protein 3 (TIM-3) is expressed on CD8+ T cells and T helper 1 (Th1) cells, serving as a potent immune inhibitor (35, 38, 49, 59). It is also detected on monocytes, dendritic cells, and macrophages (38, 60, 61). Lymphocyte-activation gene 3 (LAG-3) expression is increased on activated CD4+ T cells, CD8+ T cells, B cells, and NK cells (35, 49, 62, 63). Some studies have shown that LAG-3 and TIM-3 are strongly upregulated on T cells in COVID-19 patients (62, 64, 65). Importantly, TIM-3 and LAG-3 could be utilized to identify COVID-19 patients with bad prognoses (66–68). Furthermore, Shahbaz et al. found significant upregulations of TIM-3 on both peripheral blood CD4+ and CD8+ T cells in COVID-19 patients, compared with healthy controls (40). Other studies reported significant upregulations of TIM-3 and LAG-3 on both peripheral blood CD4+ and CD8+ T cells in severe compared with mild and moderate diseases (**Figure 1**) (40, 62). Another study observed significant elevations of soluble TIM-3 (sTIM-3) and soluble LAG-3 (sLAG-3) in severe COVID-19 patients, compared with mild disease (44). Furthermore, Chen et al. found that the plasma level of sTIM-3 was significantly higher in severe COVID-19 patients, compared with healthy controls (69). Moreover, Diao et al. showed a significant increase in TIM-3 expression on peripheral blood CD4+ T cells in COVID-19 patients, which could contribute to the functional exhaustion of these cells (67). Also, they found a correlation between TIM-3 expression and the severity of the disease in COVID-19 patients (67). In line with these findings, Modabber et al. identified higher TIM-3 expression on peripheral blood CD4+ T cells in critical COVID-19 patients than in moderate and severe diseases (60). Furthermore, some COVID-19 inpatients from Nanjing Hospital/China were evaluated, and it was found that the majority of exhausted T cells expressed LAG-3 (70). Another study found that NK cells from the majority of COVID-19 patients appeared exhausted based on the expression of LAG-3 (71). Therefore, exhaustion of these cells could be associated with serious illness and weak antiviral immune responses.

### T-Cell Immunoreceptor With Immunoglobulin and ITIM Domain

T-cell immunoreceptor with immunoglobulin and ITIM domain (TIGIT) is expressed on activated T cells, as well as NK cells, and Tregs (72–74). In severe viral diseases, the sustained expression of TIGIT in response to persistent antigen can result in T-cell exhaustion (8, 43). Shahbaz et al. found a significant upregulation of TIGIT on peripheral blood CD8+ and CD4+ T cells in COVID-19 patients, compared with controls (40). In line with these observations, TIGIT expression on peripheral blood CD8+ T cells was higher in severe compared with mild patients (**Figure 1**) (40, 43, 75). Conversely, Herrmann et al. observed no significant differences in TIGIT expression in COVID-19 patients but substantially lower than that of controls (62). In addition, Hsieh et al. found that higher frequencies of NK cell subsets expressing TIGIT eliminated the viruses faster than cells with lower levels of TIGIT in COVID-19 patients (76). Moreover, Shahbaz et al. indicated that overexpressions of TIGIT, TIM-3, and CTLA-4 were not associated with exhaustion and impairment of peripheral blood T-cell functions. More accurately, these expressions on activated T cells are to avoid harmful hyper-immune reactions (40).

### V-Domain Ig Suppressor of T-Cell Activation

V-domain Ig suppressor of T-cell activation (VISTA) is an immune checkpoint receptor that regulates T-cell function (77). It is expressed in significant levels on T cells and myeloid cells (78). In contrast to other IC receptors that are expressed after immune-cell activation, VISTA is expressed in stable conditions on both T cells and myeloid cells (79). Overexpression of VISTA leads to increase in T-cell exhaustion and reduction in their proliferation (77, 80). Some studies found that V-set and immunoglobulin domain-containing 3 (VSIG-3) is a ligand of VISTA, and its interaction can inhibit T-cell proliferation (79, 81, 82). Moreover, another study found that VISTA interacts with Gal-9 secreted by tumor cells as a ligand in acute myeloid leukemia (83).

In COVID-19, VISTA was highly expressed on peripheral blood T cells (40, 84). Shahbaz et al. reported a significant upregulation of VISTA on peripheral blood CD8+ and CD4+ T cells in COVID-19 patients, compared with controls (40). Furthermore, VISTA expression levels on T cells were found to be considerably greater in severe COVID-19 patients versus those with mild diseases (**Figure 1**) (40). Another study found that overexpression of VISTA on exhausted T cells can occur in chronic viral illnesses like COVID-19 (85). As a result, viral multiplication is likely to be uncontrollable (85).

### B- and T-Lymphocyte Attenuator

B- and T-lymphocyte attenuator (BTLA), a member of the CD28 Ig-superfamily, is structurally and functionally similar to CTLA-4 and PD-1 (86–89). BTLA is mostly expressed on B cells and both CD4+ and CD8+ T cells (90–93). Also, it can be expressed on DCs and monocytes (88). BTLA expression is reduced rapidly upon T-cell activation (89). BTLA differs from the rest of the Ig superfamily because it can bind to the herpesvirus entry mediator (HVEM), one of the TNFR superfamily members (35, 86–88). In COVID-19 patients, the BTLA was significantly elevated on peripheral blood CD4+ and CD8+ T cells, when compared to the normal group (90). This elevation of BTLA serves to counteract the initial activation of T cells (94). Another study observed a significant elevation of soluble BTLA (sBTLA) in severe COVID-19 patients, compared with mild disease (44). Moreover, Sharif-Askari et al. found a link between BTLA upregulation and COVID-19 severity (56). Moreover, Schultheiß et al. reported a significant upregulation of BTLA on both CD8+ and CD4+ T cells in COVID-19 patients, compared with healthy controls (90). In an *in vitro* study, Sumida et al. found that production of IFN-β during viral infection suppresses the expression of CD160, TIGIT, and BTLA on CD8+ and CD4+ T cells (95).

### Other Immune Checkpoints

CD244 (2B4) is expressed on T cells, as well as NK cells, DCs, and monocytes (96). Also, CD160 is expressed on T cells, NK cells, and NKT cells (96, 97). In addition, NKG2A is expressed on T cells and NK cells (98). The expression of these ICs might lead to exhaustion of CD4+ and CD8+ T cells (99–101). Some studies have linked the increased expression of these inhibitory receptors to CD8+ T-cell exhaustion in chronic viral infections such as influenza, HIV, and HCV (31, 96, 100). In COVID-19 patients, Shahbaz et al. found an overexpression of 2B4 on peripheral blood CD4+ and CD8+ T cells; however, CD160 was upregulated on CD4+ T cells but not on CD8+ T cells, compared with controls (40). Additionally, NKG2A was upregulated on peripheral blood CD4+ and not on CD8+ T cells in severe COVID-19 patients, compared with mild and moderate diseases (**Figure 1**) (40). Despite previously reported associations between T-cell dysfunction and overexpression of these inhibitory receptors in viral infections (96, 98, 100), Shahbaz et al. showed that such overexpression was associated with functional T cells against SARS-CoV-2 (40). Furthermore, Zhang et al. showed that the expression of CD160 on NKT cells was increased significantly in moderate COVID-19 patients, compared with severe illness (97). This might imply that the presence of CD160 on NKT cells improves disease control through direct cytotoxicity (97, 102). On the other hand, Zheng et al. observed an overexpression of NKG2A on exhausted NK cells and CD8+ T cells in severe COVID-19 patients (98). Therefore, the upregulation of NKG2A could be associated with functional exhaustion of cytotoxic lymphocytes at the early stage, which could result in progression of the disease (98).

### Cross Talks Between Immune Checkpoints

Some of T-cell inhibitory receptors appear to be co-expressed during exhausted T-cell differentiation. Interestingly, Yang et al. showed that PD-1 binds to the TIM-3 ligand Gal-9, which attenuates Gal-9/TIM-3-induced cell death (103). Moreover, Baitsch et al. found that naive T cells are primarily controlled by BTLA and TIM-3 receptors, whereas effector cells interact *via* larger amounts of inhibitory receptors (104). Furthermore, Okazaki et al. demonstrated that a synergistic effect was found between LAG-3 and PD-1 in the regulation of T-cell function (105). Indeed, Koyama et al. observed an upregulation of TIM-3 in tumor tissues following anti-PD-1 treatment. Consequently, adaptive resistance to anti-PD-1 therapy was acquired (106). It is possible that blocking several immune checkpoints with particular monoclonal antibodies may lead to improvements in the outcomes of various chronic viral infections, as well as in several types of cancer (107).

## Immune Checkpoint Ligands in COVID-19

Binding of IC receptors with their ligands suppresses T-cell activity and function, helping in the regulation of immunity (108). Viral infections induce the overexpression of some IC ligands in different immune cells, resulting in a decrease of the viral clearance and increased mortality (109, 110). Herein, we present the few available studies that investigated IC ligands in COVID-19 patients.

### PD-L1 and PD-L2

PD-L1 is broadly expressed on hematopoietic and non-hematopoietic cells (111). PD-L2 (also known as B7-DC) is mostly expressed in macrophages, activated DCs, Th2 cells, bone marrow-derived mast cells, and peritoneal B1 cells (112). Importantly, PD-1 and its ligands PD-L1 and PD-L2 were elevated during acute viral infections and after sustained viral infections (111). The expression of PD-L1 on basophils and eosinophils was associated with COVID-19 severity (**Figure 1**) (113). In COVID-19, dendritic cells and monocytes lack maturation markers and have elevated levels of PD-L1 (114). Moreover, Monaghan et al. reported a significant overexpression of PD-L1 and PD-L2 in peripheral blood of patients who died from COVID-19 (115). SARS-CoV-2 induced an overexpression of PD-L1 in epithelial cells, and it was dysregulated in a variety of immune cells including neutrophils, gamma delta T cells, monocytes, and CD4+ T cells of COVID-19 patients (116). These results indicate that PD-L1 has a prognostic role in COVID-19 patients (116). Blood levels of pro-inflammatory cytokines IL-6, IL-17, and IL-8 were markedly elevated in severe COVID-19 patients, together with elevated macrophage and neutrophil activity (117, 118). Therefore, overexpression of PD-L1 on the surface of immune cells in COVID-19 patients could be due to the presence of these pro-inflammatory cytokines (118). In other studies, it has been reported that PD-L1 was overexpressed on monocytes, and the plasma of COVID-19 patients contains higher levels of soluble PD-L1 (sPD-L1), compared with healthy controls (119, 120). Moreover, another study found that the serum level of sPD-L1, but not sPD-L2, was significantly higher in severe COVID-19 patients (44).

### Galectin-9

Galectin-9 (Gal-9) is a galactoside-binding protein expressed by different types of immune cells including T cells, B cells, macrophages, and mast cells, and it is involved in the regulation of overactive immune responses (121). Gal-9 is a ligand for TIM-3, and their interactions induce apoptosis and reduce T-cell activity (121). Gal-9 is significantly expressed on immune cells in viral infections, and autoimmune and malignant diseases (109). Soluble Gal-9 (sGal-9) was increased in the plasma during chronic viral disease, and it may suppress the immune activity against the viral infection (109). A recent investigation reported that circulating Gal-9 levels were elevated in humans infected with various viruses (122). These findings imply that viral infections induce Gal-9 overexpression. Schultheiß et al. reported that sGal-9 was significantly increased in severe COVID-19 patients, compared with patients after recovery and healthy controls (90). Moreover, Bozorgmehr et al. found that plasma Gal-9 concentrations were significantly greater in patients with severe COVID-19, compared with those with mild/moderate disease (**Figure 1**) (123). Plasma levels of the full-length and truncated forms of Gal-9 and Osteopontin (OPN) could serve as representative inflammatory biomarkers. In severe patients, cleavage of Gal-9 and OPN was found to be related to lung function and inflammation, but not the full length of Gal-9 and OPN (124). Therefore, the cleaved forms of OPN and Gal-9 could be useful in monitoring inflammation in COVID-19 patients with pneumonia (124, 125). In another study, COVID-19 patients were shown to have higher levels of Gal-9, Gal-3, and Gal-1, compared with healthy controls (120). Another study reported that Gal-9 was overexpressed on T cells in severe patients, compared with healthy controls (40). Gal-9 has been associated with a cytokine storm in COVID-19 (123). Furthermore, it has a positive correlation with pro-inflammatory cytokines such as IL-6 and tumor necrosis factor-α (TNF-α), suggesting that Gal-9 inhibition could be a potential therapeutic approach in COVID-19 patients (123).

### Galectin-3

Galectin-3 (Gal-3) is a galactoside-binding protein expressed by all types of immune cells (126). Gal-3 has been related to several inflammatory diseases (126). A recent study reported that Gal-3 levels in macrophages, monocytes, and dendritic cells were increased in patients with severe COVID-19, compared with mild diseases (**Figure 1**) (127). Moreover, the serum level of Gal-3 was significantly higher in severe COVID-19 patients, compared with healthy controls (69, 128). It has been reported that Gal-3 was upregulated in proliferating T cells in severe cases of COVID-19, and frequently the hyperinflammation phase involves the overexpression of Gal-3, TNF-α, and IL-6 (129). Therefore, inhibition of Gal-3 could be a helpful approach in the treatment of COVID-19 by lowering the inflammatory reaction and preventing viral adherence to host cells (126, 127, 130). Additionally, a recent study reported higher levels of Gal-3, and Gal-1 in COVID-19 patients, compared with healthy controls, implying that Gal-3 could be a useful biomarker for disease prognosis (131). Another study reported that COVID-19 patients with serum levels of Gal-3 more than 35.3 ng/ml were associated with higher mortality, ICU hospitalization, and severe acute respiratory syndrome, implying its importance as a prognostic biomarker for mortality and disease severity (132).

### B7-H3 (CD276)

B7-H3 (CD276) has both co-stimulatory and co-inhibitory roles (133). It interacts with the TLT-2 receptor to enhance T-cell activation, whereas binding to unknown receptors results in co-inhibition of T cells (133, 134). It is expressed on activated DCs, NK cells, T cells, B cells, and monocytes (135). There are very limited studies investigating B7-H3 in COVID-19 patients. A recent study reported that CD276 was upregulated in the lung during COVID-19 (136).

### CD155 and CD112

CD155 (PVR) and CD112 (PVRL2, nectin-2) have both co-stimulatory and co-inhibitory roles. Both are expressed on monocytes, and DCs (137, 138), and they are recognized by a different group of receptors expressed on T cells and NK cells, namely, DNAM-1 (CD226), TIGIT, and TACTILE (CD96) (139, 140). During the activation process, CD155 and CD112 interact with DNAM-1 to enhance NK- and T-cell activity (141). On the other hand, TIGIT interacts with these ligands to inhibit the activation of NK and T cells (72, 140–142). With regard to COVID-19, Hsieh et al. reported that SARS-CoV-2 induced the overexpression of CD155 on infected cells, which binds to its receptor TIGIT on NK cells, resulting in decreased immune responses and viral clearance (76). Additionally, Wilk et al. reported a significant expression of CD112 on monocytes of hospitalized COVID-19 patients, compared with mild disease and healthy controls (**Figure 1**) (143).

### LSECtin

Lectin (LSECtin), also known as CLEC4G, is a co-inhibitor of human T-cell immunity (144). A recent study showed that LSECtin suppresses human T-cell activation and proliferation *via* the butyrophilin family receptor BTN3A1 (144). Lu et al. reported that analysis of pulmonary cells from COVID-19 patients showed an overexpression of different C-type lectins such as L-SIGN, LSECtin, DC-SIGN, ASGR1, and CLEC10A on myeloid cells (145). Although these receptors do not promote active multiplication of SARS-CoV-2, they generate pro-inflammatory responses in myeloid cells, which are associated with COVID-19 severity (145).

## Perspective

The expression of ICs in COVID-19 patients is an important manifestation, contributing to worse clinical outcomes. Most available studies evaluated IC receptors/ligands individually or in small combinations. Comprehensive co-expression and cross talk investigations of multiple IC receptors or ligands on specific immune-cell subpopulations in COVID-19 patients are lacking. Identification of the specific immune-cell subpopulations expressing IC receptors or ligands in severe versus mild/asymptomatic COVID-19 patients is critical for prognostic purposes and therapeutic targeting. Overall, few studies investigated different receptors/ligands in the same COVID-19 patients. Based on these studies, there are some evidence supporting the use of a panel of IC receptors/ligands as prognostic biomarkers in severe COVID-19 patients; this panel could include upregulations of PD-1, CTLA-4, TIM-3, PD-L1, Gal-3, and Gal-9. Further and well-designed studies are still needed to investigate expression profiles and functions of IC receptors and ligands in severe, compared to mild and asymptomatic COVID-19 patients.

## Conclusion

COVID-19 is a pandemic disease that is impacting people all over the world. The severity of the disease is determined by the signs and symptoms that individuals exhibit. An enhanced expression of immune checkpoint molecules can result in stimulation of the apoptosis of T cells, decline in the number of T cells, and lymphopenia. Some studies reported a relationship between upregulation of IC receptors on T cells and the severity of COVID-19. Specifically, when immune cells are overactivated, ICs are upregulated and inflammatory cytokines are produced in excessive amounts, which increases the disease severity. Therefore, IC overexpression in COVID-19 patients might not be due to T-cell exhaustion with impaired antiviral responses. Some studies found that the overexpression of IC receptors on T cells may modulate the immune response and protect vital organs from an excessive inflammatory response in severe COVID-19 patients. Overexpression of some of these IC receptors can be used as prognostic biomarkers for COVID severity. Clearly, targeting inhibitory ICs should be carefully considered because the efficacy and safety of blocking inhibitory ICs in COVID-19 patients have not yet been fully elucidated.

Few studies have investigated the expression level of IC ligands in COVID-19 patients. Based on the few available studies, there is a relationship between disease severity and increased expression of IC ligands. However, there are no available studies investigating the expression levels of some IC ligands including B7-H4, B7-H5, and B7-H6 in COVID-19 patients, and it would be interesting to do that.

Cancer patients receiving immune checkpoint inhibitors (ICI) may have greater immunological competence as a consequence of their reactivated T cells. However, this may lead to an increase in the risk of cytokine release syndrome (CRS), a vital manifestation in COVID-19 patients (146, 147). Few studies found a high percentage of ICI-related CRS cases following ICI administration in cancer patients (147, 148). However, other studies found that there were no associations between administration of ICI with mortality in cancer patients with COVID-19 (149, 150). Recently, ICI could be used as a potential therapeutic approach against COVID-19 in non-cancer patients (26). The majority of the concerns regarding ICI administration are related to an increase in inflammatory cytokine secretion as a consequence of reactivated of exhausted T cells, which might lead to organ damage (147). However, another study found that organ damage in COVID-19 patients is caused by virus infection itself rather than cytokine storm (151). Additionally, Yatim et al. demonstrated that ICI therapy was not associated with severe COVID-19, rather it increases specific anti–SARS-CoV-2 T-cell immunity (152). Furthermore, another study found that the PD-1 inhibitor is able to enhance the specific T-cell immune response to SARS-CoV-2 antigens (153). In addition, TGF-β and IL-6 were upregulated in COVID-19 patients, suggesting that targeting these cytokines may improve COVID-19 outcomes (154–156).

Most of the available studies on COVID-19 patients who have undertaken ICI are concentrated on PD-1 inhibition. Other ICIs in this setting should be studied as well. More studies are needed to evaluate the safety of ICI in cancer and non-cancer COVID-19 patients.

Currently, there are different COVID-19 vaccinations including BNT162b2 (BioNTech, Pfizer), AZD1222 (Oxford, AstraZeneca), Ad26.CoV2.S (Janssen), mRNA-1273 (Moderna), BBIBP-CorV (Sinopharm), Sinovac-CoronaVac, BBV152 COVAXIN (Bharat Biotech), and NVX-CoV2373 (Covovax). Unfortunately, there are no available studies investigating IC receptors and/or ligands in individuals following any of the different COVID-19 vaccinations. Comprehensive studies are required on patients receiving COVID-19 vaccines to determine any changes in the expression and function of IC receptors and ligands on different immune cells following these vaccinations.

## Author Contributions

MA-M and AA wrote the article and prepared the figure/table. EE conceived the concept, acquired the funds, and supervised and performed the writing of the review and the editing. All authors contributed to the article and approved the submitted version.

## Conflict of Interest

The authors declare that the research was conducted in the absence of any commercial or financial relationships that could be construed as a potential conflict of interest.

## Publisher’s Note

All claims expressed in this article are solely those of the authors and do not necessarily represent those of their affiliated organizations, or those of the publisher, the editors and the reviewers. Any product that may be evaluated in this article, or claim that may be made by its manufacturer, is not guaranteed or endorsed by the publisher.

## Abbreviations

BALF, bronchoalveolar lavage fluid; BTLA, B- and T-lymphocyte attenuator; COVID-19, Coronavirus disease 2019; CTLA-4, cytotoxic T lymphocyte-associated antigen; DCs, dendritic cells; FDA, Food and Drug Administration; Gal-1, Galectin-1; Gal-3, Galectin-3; Gal-9, Galectin-9; HVEM, herpesvirus entry mediator; IC, immune checkpoint; ICU, intensive care unit; IL-6, Interleukin 6; IL8, Interleukin 8; LAG-3, lymphocyte-activation gene 3; NK, natural killer; NKT, natural killer T; OPN, Osteopontin; PD-1, programmed cell death 1; PD-L1, programmed cell death ligand 1; PD-L2, programmed death ligand 2; RdRp, RNA-dependent RNA polymerase; TGF-β1, transforming growth factor-β1; Th1, T helper; TIGIT, T-cell immunoreceptor with immunoglobulin; ITIM domain, TIM-3 T-cell immunoglobulin and mucin domain-containing protein 3; TLT-2, TREM-like transcript 2; TNFR, tumor necrosis factor receptor; TNF-α, tumor necrosis factor alpha; Treg, regulatory T cells; VISTA, V-domain Ig suppressor of T-cell activation; VSIG-3, V-set and immunoglobulin domain-containing 3.
